# Anaemia in pregnancy: prevalence and associated socio-demographic and obstetric factors in urban and rural communities in Nsukka area of Enugu State, Nigeria

**DOI:** 10.4314/ahs.v24i2.22

**Published:** 2024-06

**Authors:** Scholastica Ngozi Eze, Peace Nwanneka Ani, Cyril Onyinyechukwu Anoshirike

**Affiliations:** Department of Nutrition and Dietetics, Faculty of Agriculture, University of Nigeria, Nsukka, Nigeria

**Keywords:** Anaemia, pregnancy, women of childbearing age, Nigeria

## Abstract

**Background:**

Anaemia in pregnancy is a major cause of maternal death especially, in developing countries.

**Objectives:**

The study was aimed at determining the prevalence of anaemia and its associated socio-demographic and obstetric factors among pregnant women in Nsukka Area of Enugu State, Nigeria.

**Methods:**

Pregnant women numbering 386 participated in the study. Respondents' socio-demographic and obstetric data were collected using validated structured questionnaire. Haemoglobin concentration (Hb conc) was determined and used to categorize the women following WHO classification. Data were analyzed using descriptive statistics, Duncan's new multiple range tests, T-test and Pearson's correlation coefficient.

**Results:**

More than half (55.9%) of the respondents had low Hb conc; with 39% and 16.9% having mild and moderate anaemia, respectively. Hb conc was significantly influenced by age, occupation, educational and income levels (P<0.05). Hb conc significantly increased with increase in educational and income levels. Negative correlation existed between respondents' parity and Hb (r=-0.281; P<0.05).Women with 4 - 6 previous pregnancies had the lowest Hb conc (10.18±0.86g/dl) among the respondents.

**Conclusion:**

Anaemia in pregnancy is highly prevalent in Nsukka, and was associated with younger age, low educational and income levels, and higher parity. Girl-child/women's education must be highly prioritized, and adolescent marriage/pregnancy prevented through community-based approaches.

## Introduction

Anaemia in pregnancy is defined as haemoglobin concentration of less than 11g/dl in venous blood. It increases perinatal risks for both mother and neonate. It is associated with increased maternal morbidity and mortality. The risk of haemorrhage, the commonest cause of maternal death is increased by anaemia in pregnancy;^1^ hence, a moderate haemorrhage can be fatal in an anaemic woman. On the part of the foetus, anaemia in pregnancy causes low birth weight and preterm births, and increases overall infant mortality.

Although, anaemia in pregnancy is a global public health problem, it is more common in developing countries than in developed countries.^2^ The prevalence of anaemia among pregnant women in Africa ranged from 47% in East Africa to 56% in West Africa.^3^ Anaemia in pregnancy is a major contributing factor to maternal mortality in developing world.^4^,^5^ WHO estimated that more than half of global maternal deaths occur in Sub-Saharan Africa. In Nigeria, approximately 58,000 women died in 2015 from pregnancy related complications, representing nearly 20% of global maternal deaths. A woman's chance of dying from pregnancy and childbirth in Nigeria is in a ratio of 1: 22.^2^

Pregnant women are vulnerable to anaemia due to plasma volume expansion (which leads to haemodilution), increased nutrient demands for formation of foetal organs and rapid growth of the foetus, coupled with social and biological stresses faced by women during pregnancy. It has been shown that women simultaneously exercise roles in reproduction, economic production and home production, often with damaging consequences for their own nutritional status.^6^

Many predisposing factors to anaemia in pregnancy are preventable, but there is need for baseline prevalence data to create awareness of its magnitude in our environment in order to formulate strategies to reduce its occurrence as well as its adverse health consequences. Information on the prevalence would also be useful for managers of health institutions and for district, provincial, and national maternal, child, and women's health programme development.^7^ The study was therefore, aimed at determining the prevalence and associated socio-economic and obstetric factors of anaemia in pregnancy in Nsukka Area in Nigeria as a preliminary study with the view to build health promotion activities that would alleviate identified problems. It became pertinent due to the fact that it had been observed that there are abundant sources of micronutrient-rich indigenous foods in some areas and people in these areas still suffer deficiencies of the micronutrients. Nsukka Area is known to produce a lot of food crops and rear animals such as poultry, pigs and goats, but not much is consumed by local people because they sell most of their products to outside communities as source of income.^8^

## Methods

The study was carried out in Nsukka town (urban), Ede-Oballa and Okpuje (rural communities) in Nsukka Area of Enugu State, Nigeria. Two health facilities with highest enrolment of pregnant women for antenatal care in each of the communities were selected using purposive sampling method. The respondents (386 pregnant women) were randomly selected from women who were attending antenatal clinics in the six selected health facilities.

### Ethical considerations

Ethical approval was obtained from Ethical Committee of Enugu State Ministry of Health prior to the study. Informed consent was obtained from the women in a culturally appropriate manner after thorough explanation of what the study entailed and its purpose, with assurance of confidentiality of all information collected. Only those who had no apparent sign of ill-health and who gave their consent participated in the study.

### Inclusion and exclusion criteria

Only pregnant women who gave their consent and had no apparent signs of ill- health were included in the study. Those who were sick and those with known health problems such as SS genotype which could predispose them to anaemia were not included in the study.

### Data collection

Instruments used for data collection were questionnaire and biochemical analysis of blood samples.

### Questionnaire

A validated structured questionnaire was used to collect data on socio-demographic and obstetric characteristics of the respondents. The questionnaire was validated by Lecturers in the Department of Nutrition and Dietetics, University of Nigeria, Nsukka. Each item of the questionnaire was explained clearly to the respondents in the language they understood and responses of illiterate respondents were obtained through interview and recorded accordingly.

### Biochemical analysis of blood samples

Blood samples were collected from the respondents for determination of haemoglobin concentrations. The blood samples were carefully collected in non-fasting state and haemoglobin concentrations were determined by cyanmethemoglobin method.^9^ The respondents were categorized according to their haemoglobin values following WHO classification of anaemia.^10^

### Statistical analysis

All analyses were conducted using the Statistical Package for the Social Sciences software, version 22. Duncan's new multiple range tests and t-test were used to separate group means, and compare the means, respectively. We used Pearson's correlation coefficient to test relationship between variables. Significant differences were judged at P<0.05.

## Results

Table 1 presents the distribution of respondents by age, parity, age at first childbirth, and location. The table shows that higher proportion (53.1%) of the respondents was aged 26 - 35 years, and some respondents (5.4%) were below 20 years. It was shown that respondents below 20years and those aged 20 - 25 years were more among the rural (7.4% and 31.1%, respectively) than urban (1.6% and 19.4%, respectively) respondents. Higher percentage of the respondents (49%) had 1 – 3 childbirth prior to the current pregnancy. Approximately 21% were primigravidae, and 10.1% of the respondents had more than 6 childbirths. The respondents that had 4 - 6 and more than 6 childbirths prior to the current pregnancy were much more in rural (24.9% and 12.8%, respectively) than in urban (10.8% and 4.7%, respectively) areas.

**Table 1:** Distribution of respondents according to age, parity, age at first childbirth, and location

Characteristics	Urban		Rural		Total	
	F	%	F	%	F	%
***Age of respondents***						
< 20years	2	1.6	19	7.4	21	5.4
20 – 25years	25	19.4	80	31.1	105	27.2
26 – 35years	89	69.0	116	45.1	205	53.1
36 – 45years	13	10.1	42	16.3	55	14.2
**Total**	**129**	**100**	**257**	**100**	**386**	**100**
***Parity***						
Primigravidae	32	24.8	48	18.7	80	20.7
1 – 3	77	59.7	112	43.6	189	49.0
4 - 6	17	10.8	64	24.9	78	20.2
>6	6	4.7	33	12.8	39	10.1
**Total**	**129**	**100**	**257**	**100**	**386**	**100**
***Age at 1^st^ childbirth***						
< 20years	16	12.4	53	20.6	69	17.9
20 – 25years	39	30.2	124	48.3	163	42.2
26 – 35years	69	53.5	72	28.0	141	36.5
36 – 45years	5	3.9	8	3.1	13	3.4
**Total**	**129**	**100**	**257**	**100**	**386**	**100**

Key: F: frequency

%: percentage

**Figure 1:**
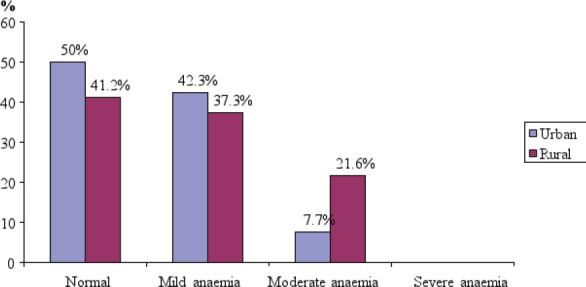
Percentage distribution of haemoglobin concentration of respondents by location

The distribution of the respondents according to Hb conc is presented in Table 2. The table shows that less than half (44.1%) of the respondents had normal Hb conc (11g/dl and above). The other 39.0% and 16.9% of the respondents, respectively had mild (Hb: 10.1 - 10.9g/dl) and moderate (Hb: 7 – 10g/dl) anaemia. The percentage distribution of respondents' Hb conc by location is presented in Figure 1. The percentage of the respondents who had mild and moderate anaemia were higher in the rural (58.9%) than in the urban area (50%).

**Table 2:** Distribution of the respondents according to haemoglobin concentrations

Haemoglobin concentration (g/dl)	Frequency	Percentage (%)
Normal (11 & above)	170	44.1
Mild anaemia (10.1 – 10.9)	151	39.0
Moderate anaemia (7 – 10)	65	16.9
Severe anaemia (< 7)	-	-
**Total**	**386**	**100**

Table 3 presents the respondents' mean Hb conc by educational and income levels, age and occupation. It was shown that the women's Hb conc were significantly influenced by their educational and income levels, age and occupation (P<0.05). The higher the respondents' educational level the higher their Hb conc. The respondents' Hb conc also increased significantly with increase in income level. It was shown that respondents below the age of 20 years had significantly lowest Hb conc (8.5g/dl). Artisans and farmers had significantly lowest mean Hb conc (9.89g/dl and 10.1g/dl, respectively), followed by traders (10.68g/dl).

**Table 3:** Respondents' mean Hb concentration by educational and income levels, age and occupation

Variables	Hb conc. (g/dl)	F-value	P-value
***Educational level***			
< Secondary	10.48^b^ ±1.07	3.676	0.030
Secondary	10.97^ab^0.91		
Tertiary	11.50^a^±0.89		
***Income level***			
Low (<₦20,000)	10.19^b^±0.76	8.039	0.001
Medium	11.03^a^±0.99		
(₦20,000 - 50,000)			
High (>(₦50,000)	11.47^a^±0.64		
***Age (years)***			
< 20	8.50^b^±0.00	**5.870**	**0.001**
20 – 25	10.98^a^±0.85		
26 – 35	11.03^a^±0.94		
36 – 45	10.38^a^±1.02		
***Occupation***			
Artisans	9.89^c^±0.86	4.295	0.002
Civil servants	11.38^a^ ±1.02		
Farmers	10.10^bc^±0.69		
Unemployed	11.29^a^± 0.94		
Students	11.01^ab^±1.44		
Traders	10.68^abc^±0.51		

Key: ^abc^means with similar superscripts in the same column for each characteristic are not significantly different (P > 0.05) from each other.

The respondents' mean Hb conc by parity and trimester are presented in Table 4. There was no statistical difference in mean Hb values with regard to parity and trimester, although, Hb conc was lowest (10.18g/dl) among women with 4 - 6 previous pregnancies, and lower (10.70g/dl) in women in second trimester than those in third trimester (10.92g/dl). Table 5 shows negative relationship between the respondents' parity and their Hb conc (r = - 0.281; P<0.05).

**Table 4:** Mean haemoglobin concentrations of respondents by parity and trimester

Variables	Hb conc (g/dl)	F-value	P-value
***Parity***			
0	11.05^a^±1.13	**2.470**	0.069
1 to 3	10.99^a^±0.93		
4 to 6	10.18^a^±0.86		
> 6	10.90^a^±0.00		
***Trimester***			
Second trimester	10.70^a^±1.12	0.713	0.490
Third trimester	10.92^a^±0.97		

Key: ^ab:^ means with similar superscripts in the same column for each characteristic are not significantly different (P > 0.05) from each other.

**Table 5:** Results of correlation between variables

		Age (years)	Parity	Hb (g/dl)	Trimester
**Age (years)**	Pearson's correlation	1	.695**	.009	.084
	P value		.000	.938	.469
**Parity**	Pearson's correlation		1	-.281*	-.065
	P value			.013	.577
**Hb (g/dl)**	Pearson's correlation			1	.125
	P value				.280
**Trimester**	Pearson's correlation				1

** Correlation is significant at 0.01 level (2-tailed)

* Correlation is significant at 0.05 level (2-tailed)

## Discussion

There was high (55.9%)prevalence of anaemia among the study population and this was in line with many earlier reports from developing countries.^3^,^11^-^13^ Earlier studies showed that the prevalence of anaemia in pregnancy in developing countries ranged from 35.0% to 75.0%^13^, 47% in East Africa and 56% in West Africa.^3^ It is obvious from these results that the situation has not improved more than two decades after the prevalence in West Africa was reported in the year 2000 to be 56%^6^. Anaemia in pregnancy elevates the risks of haemorrhage and death during childbirth^1^,^14^ and severe anaemia is an associated cause in 50% and the main cause in up to 20% of maternal deaths in developing countries.^15^ The prevalence of anaemia among the study population was higher in the rural (58.9%) than in the urban area (50%). This is consistent with the findings of a recent study in Anambra State.^16^ which found a higher prevalence of anaemia among women of childbearing age in riverine rural areas (73.3%) than in urban areas (53.3%). This has been attributed to a number of factors, including inadequate intake and the sale of micronutrient-rich foods harvested by these rural women to generate income for other household needs.^16^ The high prevalence of anaemia among this study population was associated with low educational and income levels, occupation, young age and higher parity. The respondents' Hb conc decreased with decrease in both educational and income levels. The influence of educational levels on the women's Hb conc was a function of lack of awareness of importance and constituents of adequate diet. It was earlier observed that the dearth of knowledge of health and economic benefits of locally available foods precipitated faulty food choices and habits.^17^,^18^ Again, education is linked with income which also influenced the Hb conc of the women. It is known that the nature, quality and quantity of foods consumed are functions of income. Socio-economic status determined purchasing power and thus influenced the quality and quantity of diet consumed.^17^ Earlier reports showed that low income families were not able to purchase food items rich in haem iron such as beef, organ meat and egg which have higher bioavailability.^8^,^18^ Even home reared animals such as poultry, pigs and goats are not often consumed by local people because they sell most of them to outside communities as source of income for other needs.^8^ Economic power was observed to be the most important determinant of women's relative equality, which affects decision making, life style options, and control over resources such as food.^6^ Socio-economic status of women also affects their health seeking behaviour.^13^ These emphasize the important role of economic empowerment of women in reducing prevalence of anaemia in Nigeria.

The higher Hb conc of civil servants and students than artisans, farmers and traders was a function of education. Artisans, farmers and traders might not have had as much nutrition education as their educated counterparts. The higher Hb conc of the unemployed is attributed to the fact that in addition to nutrition education and purchasing power, proper feeding demands time for meal preparation and consumption. It was earlier reported that time constraint was a serious problem among house wives who worked long hours outside the home.^17^ Time constraints may lead to infrequent meals, and exhaustion from work may lead to reduced appetite, all of which result in reduced overall intake and lower intake of individual nutrients.

The significantly lowest Hb conc (8.5g/dl) of the teenagers gave credence to an earlier observation that adolescents had poor eating habits and were known for snacking on low micronutrient dense foods.^17^ Despite the campaign against early marriage and teenage pregnancy, teenagers constituted 7.3% of the rural respondents and the respondents who were below the age of 20 years and those 20-25 years were higher in the rural areas (7.3% and 31.1%, respectively) than in the. urban area (1.6% and 19.4%, respectively). These results indicate that rural women married at earlier ages than urban women. The low age (<20years) of 12.4% of urban and 20.6% of rural respondents at first childbirth confirmed earlier report that early marriage and teenage pregnancy were prevalent practices in Nigeria.^8^

It is well known that pregnancy during the adolescent years constitutes an additional risk for mother and child^6^ because teenager's physical growth demands for nutrients compete with those of the growing foetus. It is known that growth spurt begins slowly for girls by the age of 14 years, but linear growth of long bones is not complete until the age of 18 years. The peak bone mass is not achieved until the age of 25 years. The development of the pelvic bones is slower than that of height and does not reach mature size until about 2 to 3 years after linear growth has ceased, thus the pelvis of the teenager that may not be fully developed increases risk of obstetric complications. Early/teenage pregnancy therefore, stunts the girl's height and precipitates much incidence of low birth weight and neonatal mortality.^20^

The negative correlation between the respondents' parity and Hb conc, and the lowest Hb conc (10.18g/dl) of multiparous women (4 - 6 previous pregnancies) agreed with an earlier report^21^ and was attributed to repeated drain on iron stores. It has been shown that the chronic under nutrition and maternal depletion following closely spaced pregnancies not only precipitates poorer nutritional status for women and their offspring, it accelerates aging and poorer health status of women. The increased biological stress associated with frequent and prolonged childbearing is accompanied by social stress for the mother due to increased family size.^14^

Data on respondents' parity suggested that family planning was not fully adopted by many families, especially in the rural areas where there were more (12.8%) women who had more than 6 previous childbirths than in the urban area (4.7%). In addition to ignorance, belief and misconceptions, early marriage/teenage pregnancy and closely spaced pregnancies are shown to promote high number of childbirths.^22^ Early marriage exposed women to frequent and prolonged childbearing.^6^ Reduction in number of pregnancies/childbirth could be achieved by beginning reproduction later, spacing conceptions to allow a minimum period of 6 months “recuperation” following complete weaning of the previous child, and ending reproduction earlier.^22^ To promote family planning which is shown to have much influence on maternal and infant mortality,^23^ it was suggested that education on the value of family planning should be targeted towards men because men often would want more children than their spouses.^24^

The lower Hb conc of women in second trimester than those in third trimester was at variance with an earlier report of WHO in which anaemia prevalence was significantly higher in 3^rd^ trimester of pregnancy than in first two trimesters.^25^ However, the results of this study confirmed another report^26^ and was attributed to the fact that haemodilution is at its peak in second trimester.

## Conclusion

Anaemia in pregnancy is prevalent in Nsukka area and is associated with younger age, low educational and income levels, and higher parity. Results of this study showed that women's nutritional and health status hinge on improved educational and economic empowerment. Women's education must therefore, be highly prioritized and facilitated by creating awareness of importance of girl-child education, free education up to secondary school level, and adult education for illiterate adult women. Community-based approaches should be used to reduce the prevalence of adolescent marriage/pregnancy and for promotion of girl-child education. More income generating activities/entrepreneurial skills for women are imperative to increase women's income and boost their spending on family food. These become even more pertinent when one considers that women play a major role in determining what their families eat. When women are healthy, educationally and economically empowered, their children thrive better and their entire families, communities and nations flourish and yield multiple dividends.
